# The Effect of Desensitizing Toothpastes and Coffee Staining on the Optical Properties of Natural Teeth and Microhybrid Resin Composites: An In-Vitro Study

**DOI:** 10.1155/2018/9673562

**Published:** 2018-12-23

**Authors:** Sedanur Turgut, Hamiyet Kılınç, Kıvanç Utku Ulusoy, Bora Bagis

**Affiliations:** ^1^Ph.D., DDS, Assoc. Professor Dr., Department of Prosthodontics, Faculty of Dentistry, Karadeniz Technical University, Trabzon, Turkey; ^2^Ph.D., DDS, Assist. Professor Dr., Department of Prosthodontics, Faculty of Dentistry, Bolu Abant Izzet Baysal University, Bolu, Turkey; ^3^Ph.D., DDS, Private Practice, Antalya, Turkey; ^4^Ph.D., DDS, Professor Dr., Department of Prosthodontics, Faculty of Dentistry, Izmir Katip Celebi University, Izmir, Turkey

## Abstract

**Objective:**

To evaluate the effect of different desensitizing toothpastes and coffee staining on the discoloration rate of natural human tooth and composite materials.

**Materials and Methods:**

A total of 56 human teeth and 56 composite specimens were used. After initial color measurements were taken, specimens and teeth were exposed to simulated toothbrushing with six desensitizing toothpastes containing different active ingredients: stannous fluoride, strontium acetate, potassium citrate and hydroxyapatite, cetylpyridinium chloride, arginine, and novamin. Specimens were then exposed to coffee staining. Throughout the staining procedure, the storing solution was refreshed every day and the specimens were brushed with the tested toothpastes. Color measurements and changes were recorded at each stage and analyzed with one-way ANOVA, Dunnett tests, and paired sample t-test (p<0.05).

**Results:**

The largest color change was obtained with the stannous fluoride toothpaste after coffee staining for natural teeth (2.6 Δ*E*_00_^⁎^) and composite specimens (3.1 Δ*E*_00_^⁎^). Coffee staining resulted in significant changes for Δ*E*_00_^⁎^ values of all groups, except for natural teeth brushed with a novamin-based (p= 0.06) toothpaste. For composite specimens, only the stannous fluoride and cetylpyridinium chloride-based toothpastes resulted in significant color changes relative to the control group. ΔL^⁎^ and Δb^⁎^ values were also increased after staining for all groups (p<0.05).

**Conclusions:**

Desensitizing toothpastes alone did not cause perceptible color changes; however, in combination with coffee staining, it tended to increased discoloration for both composites and natural teeth.

## 1. Introduction

Tooth hypersensitivity is a multifactorial condition typically characterized by sharp and brief arising in response to evaporative, thermal, osmotic, tactile, or chemical stimuli that cannot be ascribed to any other form of dental defect or disease [1]. This clinically frequent oral health problem can cause pain while breathing, eating, or toothbrushing and its prevalence has been measured as ranging from 3% to 73% [2]. The most commonly supported explanation of hypersensitivity is the hydrodynamic theory suggested by Brannstrom, which assumes that painful stimulation increases fluid flow within the dentinal tubules, stimulating baroreceptors and thus causing a neural signal and a painful sensation [3]. Therefore, ideal treatment for hypersensitivity should block the exposed dentinal tubules or reduce fluid flow within the dentinal tubules [4]. Generally accepted treatments are the application of a dentin sealer such as composite resin, glass ionomer, or the use of potassium salts, calcium phosphate, fluorides, strontium, oxalates, glutaraldehyde, and formaldehyde as tubule plugs [5]. Alternative treatments are laser treatment or mucogingival plastic surgery [2]. Sensitivity-relief toothpaste offers a convenient, easy-to-use, cost-effective, and noninvasive method of healing sensitivity. Various sensitivity-relief toothpastes are available worldwide, which include as active agents sodium fluoride (NaF), stannous fluoride (SnF_2_), arginine, potassium, strontium, or sodium calcium phosphosilicate [6, 7].

Natural teeth and restorative materials suffer staining due to both extrinsic and intrinsic factors. Extrinsic factors mainly include biofilm accumulation, smoking, or consumption of staining beverages [8]. The oral environment is exposed daily to a variety of media that have the potential to cause discoloration, resulting in esthetic problems. Many studies have examined the discoloration effects of various factors [9]. Moreover, despite the importance of toothbrushing for optimal oral hygiene, the discoloration effect of brushing on the dental structures has been noted [10, 11]. Although extrinsic stains can be removed partially or totally from the dental surface by toothbrushing [11, 12], the abrasion caused by brushing may result in gradual loss of enamel, dentin, and restorative materials [13–18], which can increase the surface roughness resulting to become more susceptible to staining [19–21].

Discoloration can be evaluated visually or with color-measuring devices such as colorimeters, which can provide accurate numerical color data [22]. Color difference (ΔE) indicates whether a change in overall shade is able to be perceived by a human eye [23]. Sharma has calculated the CIEDE2000 color difference (Δ*E*_00_^*∗*^) by using an Excel spreadsheet implementation [24]. This formula was found considerably more sophisticated, providing better indicators of human perceptibility and acceptability of color differences, and computationally involved than the equations for CIELab Δ*E*^*∗*^*ab* and the CIE94 color difference [23–25]. The CIEDE2000 formula includes not only lightness, chroma, and hue weighting functions but also an interactive term between chroma and hue differences and seems to offer improvements over the CIELab formula that would imply better clinical relevance [20]. Perceptibility threshold (PT) and acceptability threshold (AT) are major thresholds for evaluating color differences and serve as a control to assess the dental materials success and interpret visual and instrumental findings as reported by Pravina et al. [23] The PT represents the judgment of the presence/absence of exact match, with a color difference at or below it being a nearly perfect color match. However, the PT is of limited practical relevance as industry rarely strive or need to achieve an exact match, which would be time consuming and expensive. The AT defines the overall acceptance of color match/mismatch, representing the industry tolerance between the PT and the unacceptable mismatch. Consequently, an acceptable match in dentistry is a color difference at or below the AT [23]. In the previous studies, the Δ*E*_00_^*∗*^ values were compared with 50:50% PT and 50:50% AT values [23, 25–27].

Although the effect of toothbrushing on surface deterioration, roughness, and color changes have been studied [28, 29], the effect of sensitivity-relief toothpastes on staining of the natural human tooth and esthetic restorative materials has not been reported. The objectives of this study were to assess the effect of commonly used desensitizing toothpastes containing different ingredients on the optical properties of natural tooth and resin composites after immersion in coffee over a six-month period. The null hypotheses were that brushing with desensitizing toothpastes would not cause staining and would not affect the optical properties of natural tooth and resin composite materials after submitting to coffee staining.

## 2. Materials and Methods

### 2.1. Preparation of Specimens

For the resin composite group, a total of 56 disk-shaped specimens were obtained, using a brass mold with a socket (10 ± 0.05 mm diameter and 2 ± 0.05 mm thickness). The microhybrid composite (Gradia Direct X, GC) material was manipulated and polymerized by covering the top surface with a glass slide and light curing (Elipar FreeLight 2, 3MESPE) for 20 s perpendicularly. After removal from the mold, the specimens were light-cured from the bottom surface using the same parameters. Then, specimens were polished with silicon carbide (SiC) papers (Norton abrasives, Guarulhos) of decreasing abrasiveness (#600, #1200, and #2000 grit) for 10 sec each by one clinician. The specimens were measured at 3 points with a digital micrometer to ensure a standard thickness and if necessary they were reproduced. The specimens were divided into seven groups randomly and stored at 37°C and 100% relative humidity for 24 hours before initial color measurements.

For the natural tooth group, a total of 56 human central incisors, recently extracted for periodontal reasons, were selected (use of natural teeth required ethics approval, vote number 2015/57). The teeth were stored in a 0.1% thymol solution at room temperature for up to 4 weeks after extraction. Teeth with any visible caries, cracks, or hypoplastic defects were excluded. All teeth were gently polished by one clinician with a rubber cup and polishing paste (CleanPolish, Kerr) under running water for two minutes. The crowns of the teeth were then sectioned 2 mm apically to the cementoenamel junction, using double-faced diamond discs under running water (KG Sorensen, Barueri). The pulp tissues of the crown were removed with hand instruments, cleaned, and then divided into seven experimental procedure groups (n= 8). Before the baseline measurements were taken, specimens were immersed in artificial saliva at 37°C for 24 hours. The artificial saliva was prepared according to the following formula: 4.3 g xylitol, 1 g sodium carboxymethylcellulose, 5 mg calcium chloride, 40 mg potassium, 0.1 g potassium chloride, phosphate, 1 mg potassium thiocyanate, and 100 g deionized water.

### 2.2. Color Measurement

The Commission Internationale de l'Eclairage (CIE) color measurements were obtained with a colorimeter (ShadeNCC, Shofu) in a viewing booth, under D65 standard illumination based on ISO standards (ISO 7491). The colorimeter was calibrated according to the manufacturer instructions before the experimental measurements and the probe tip was positioned perpendicular in the middle of each specimen, according to a previous article [14]. The L^*∗*^a^*∗*^b^*∗*^ color notation of each composite and tooth were measured consecutively three times against a white background (*L*^*∗*^=96.68, *a*^*∗*^=−0.18, *b*^*∗*^=−0.22), and the average of the readings calculated [30]. The L^*∗*^ value measures the lightness or brightness of a material; a^*∗*^ is a measure of greenness (negative) or redness (positive); and b^*∗*^ is a measure of blueness (negative) or yellowness (positive) [31]. After the simulated tooth brushing and discoloration procedure, color coordinates of the specimens were again measured, under the same conditions as before. The Δ*E*_00_^*∗*^ values were calculated from the CIELab color space. Given a pair of color values in CIELab color space L_0_^*∗*^, a_0_^*∗*^, b_0_^*∗*^ and L_1_^*∗*^, a_1_^*∗*^, b_1_^*∗*^, and color differences were calculated with the following formula;

(1) $$\begin{array}{c} \Delta {E^{*}}_{00}=\sqrt{{\left(\frac{\Delta L^{\prime }}{kLSL}\right)}^{2}+{\left(\frac{\Delta C^{\prime }}{kCSC}\right)}^{2}+{\left(\frac{\Delta H^{\prime }}{kHSH}\right)}^{2}+RT{\left(\frac{\Delta C^{\prime }}{kCSC}\right)}^{2}{\left(\frac{\Delta H^{\prime }}{kHSH}\right)}^{2}} \end{array}$$

For a pair of specimens, ΔC' and ΔH' are the differences in chroma and hue.* S*_L_,* S*_C_, and* S*_H_ are the weighting functions for the lightness, chroma, and hue; and* k*_L_,* k*_C_, and* k*_H_ are the parametric weighting factors for variations in experimental conditions. RT is a rotation function and applied to account for the interaction between hue and chroma differences in the blue region. The parametric factors were set to 1 [24].

### 2.3. Simulated Toothbrushing

After the initial color measurements were taken, the labial surfaces of the natural teeth and the composite surfaces were exposed to simulated toothbrushing using an electronic toothbrush (Braun Oral-B Advance Power) and soft toothbrushes (Oral-B Sensitive Clean, Procter & Gamble). Specimens were fixed horizontally face-side-up, and each brush set to result in a final weight of 200 g. The toothpaste slurry was prepared in a proportion of 1:1 by weight (150 g of toothpaste and 150 ml of artificial saliva). Six different desensitizing toothpastes were used for the present study and no toothpaste was used for the control (C) group (Table 1). Artificial saliva was prepared according to the formula described in the preparation of specimens section above. Each specimen was brushed daily using 40 strokes. This number was based on an estimate that a tooth is brushed for 10 seconds in each daily toothbrushing of 2 minutes [10]. Considering that toothbrushing is performed twice a day, this means that each tooth will be submitted, on average, to 280 strokes weekly (2 strokes per second/1120 strokes in a mounth), resulting 6720 strokes for 6 months. After every 1200 strokes, brushes and toothpastes were replaced and freshly mixed toothpaste slurries applied. This cycle was repeated for each toothpaste.

Specimens were then taken from the sample holders, washed for 1 minute with an air/water spray, and put in an ultrasonic cleaner for 10 minutes, after which they were wiped dry with tissue paper. The specimens were re-evaluated for their color measurements, and color changes (Δ*E*_00_^*∗*^_1_) were recorded.

### 2.4. Discoloration Procedure

After the simulated toothbrushing and measurement of color values, the specimens were exposed to staining. The staining solution was prepared by adding 7.5 g coffee (Nescafé Classic, Nestlé) to 500 ml boiling distilled water. All specimens were immersed in the coffee solution in a stainless steel container, in a dark environment and stored at 37°C for seven days to simulate intra-oral conditions. Throughout the experiment, every day the staining solution was refreshed and the specimens were brushed with the study toothpastes. After the discoloration procedure, each specimen was washed and dried. Color measurements were taken for each specimen under the process described above, and the data were recorded as L_2_^*∗*^, a_2_^*∗*^ and b_2_^*∗*^. Color changes (Δ*E*_00_^*∗*^_2_) were calculated using these new values and the original values (L_0_^*∗*^, a_0_^*∗*^ and b_0_^*∗*^) (Table 2).

### 2.5. Statistical Analysis

Statistical analyses were performed with SPSS for Windows 17.0 (SPSS Inc., Chicago, IL, USA). Statistical analyses were performed with SPSS for Windows 17.0. Shapiro wilk test was used for normality of distribution. Paired sample t-test was used for evaluating tested variables (Δ*E*_00_^*∗*^_1_-Δ*E*_00_^*∗*^_2_, ΔL^*∗*^_1_- ΔL^*∗*^_2_, Δa^*∗*^_1_- Δa^*∗*^_2_, and Δb^*∗*^_1_-  Δb^*∗*^_2_). One-way analysis of variance (ANOVA) and Dunnett's tests were used for comparing the experimental groups with the control group separately for individual materials (natural teeth and composites). p<0.05 was set as significance level.

## 3. Results

Evaluating the ΔE_00_^*∗*^_1_, ΔE_00_^*∗*^_2_, ΔL_1_^*∗*^, ΔL_2_^*∗*^, Δa_1_^*∗*^, Δa_2_^*∗*^, Δb_1_^*∗*^, and Δb_2_^*∗*^ values, the results of the one-way ANOVA and Dunnett's tests indicated that there were significant differences for both natural teeth and the composite groups after coffee staining. The means, standard deviations (SD) and statistical significance of ΔE_00_^*∗*^_1_, ΔE_00_^*∗*^_2_, ΔL_1_^*∗*^, ΔL_2_^*∗*^, Δa_1_^*∗*^, Δa_2_^*∗*^, Δb_1_^*∗*^, and Δb_2_^*∗*^ values are shown in Tables 3 and 4.

For natural teeth, the mean values of ΔE_00_^*∗*^_1_ ranged between 0.3 and 1.1, while ΔE_00_^*∗*^_2_ values ranged between 1.0 and 2.6. The highest ΔE^*∗*^ was obtained by Group SF after coffee staining (ΔE_00_^*∗*^_2_=2.6). There were significant differences between the ΔE_00_^*∗*^_1_ and ΔE_00_^*∗*^_2_ values for all groups (p<0.05), except Group N (p= 0.06). Control group resulted in significantly lower ΔE_00_^*∗*^_1_ value than other groups. Evaluating the ΔE_00_^*∗*^_2_ values with the control group no significant differences were found for Group N (p=0.09). ΔL_2_^*∗*^ and Δb_2_^*∗*^ values of all groups were significantly higher than ΔL_1_^*∗*^ and Δb_1_^*∗*^ values. For Δa_1_^*∗*^, there were no significant differences for Group SF (p=0.207) and Group PcH (p=0.06).

For composite specimens, the mean values of ΔE_00_^*∗*^_1_ ranged between 0.6 and 1.6, while ΔE_00_^*∗*^_2_ values ranged between 2.1 and 3.1. Again, the highest color change was obtained by Group SF after coffee staining (ΔE_00_^*∗*^_2_=3.1). Significant differences were also found between ΔE_00_^*∗*^_1_ and ΔE_00_^*∗*^_2_ for all experimental groups (p<0.05). For all experimental procedures, only Group SF (p=0.003 for ΔE_00_^*∗*^_1_; p<0.001 for ΔE_00_^*∗*^_2_) and Group CPC (p=0.079 for ΔE_00_^*∗*^_1_; p<0.602 for ΔE_00_^*∗*^_2_) resulted in significant color changes relative to the control group.

Paired sample t-test also showed that ΔE_00_^*∗*^, ΔL^*∗*^, and Δb^*∗*^ values increased after coffee staining for all groups and these increments were statistically significant (p<0.05). There were only no significant differences for Δa_1_^*∗*^- Δa_2_^*∗*^ of Group SF for composites (p= 0.207) and Group PcH for natural teeth (p=0.06).

All groups recorded negative ∆L^*∗*^ values and positive ∆a^*∗*^ and ∆b^*∗*^ values (L^*∗*^ values decreased and a^*∗*^ and b^*∗*^ values increased) except Group N and Group C-Group N recorded positive ∆L_1_^*∗*^ and negative ∆b_1_^*∗*^ values (L^*∗*^ values increased and b^*∗*^ values decreased) for natural teeth after simulating brushing, while Group C recorded positive ∆L_1_^*∗*^ and negative ∆b_1_^*∗*^ values (L^*∗*^ values increased and b^*∗*^ values decreased) for both natural teeth and composite specimens.

## 4. Discussion

The first null hypothesis of the study was partially rejected: significant differences were found between the color change values of natural teeth and composite materials after using desensitizing toothpastes for six months. The second null hypothesis that desensitizing toothpastes would not affect stainability after coffee exposure was partially rejected for natural teeth (Group N did not show significant color changes relative to the control group) and rejected for composite materials. For all composite groups, coffee staining increased the ΔE_00_^*∗*^ values significantly. Color changes were slighter for natural teeth, for all evaluated groups.

As reported in a previous study [32], the different tolerance could be expected when compared with the color differences derived mostly from the differences in chroma. Several studies [23, 25–27] determined that 50:50% PT ranged from 0.80 to 1.30 ΔE_00_^*∗*^ units and that 50:50% AT ranged from 1.80 to 2.25 ΔE_00_^*∗*^. Paravina et al. [23] have found 50:50% PT and ATs were significantly different. CIEDE2000 50:50% PT was 0.8 ΔE_00_^*∗*^, whereas 50:50% AT was 1.8 ΔE_00_^*∗*^. In this study, the color differences were beyond those values of 50:50% PT for both natural teeth and composites, after simulated brushing and coffee staining. Evaluating the 50:50% AT, Groups SF, SA, and CPC exhibited higher ΔE_00_^*∗*^ values for natural teeth. However, for composites none of the groups were below the values of 50:50% AT.

Several in vitro studies have shown that topically applied fluoride agents may cause volume loss and surface changes in restorative materials, such as resin or ceramics [13, 33]. High fluoride concentrations have been shown to cause more significant surface damage. The most common fluoride agents currently in use are 0.4% SnF_2_ and 1.0% NaF gel. 0.4% SnF_2_ concentration has a pH of 3.2, making it more acidic than 1.0% NaF and this low pH may cause surface deterioration [33]. One study found that NaF caused less surface deterioration and discoloration for porcelain than SnF_2_ after one year of clinical exposure [33]. Other researchs have also reported that stannous formulations have a possible staining side effect [19, 20]. Significant tooth staining may be the result of stannous fluoride that generally causes products to be unstable. Hence, this ingredient is not favored for use in bleaching agents [6].

Therefore, the fact that Group SF experienced the highest discoloration in this study might be attributable to possible damage of the surfaces of both natural teeth and composites. The discoloration also may occur as a result of using the SnF_2_ formulation.

In this study, both natural teeth and the composite specimens that were exposed to simulated brushing with desensitizing toothpastes and coffee became darker and more yellow. Only novamin-containing toothpaste caused both natural teeth and the composites to become brighter (i.e., increased the L^*∗*^ value). Novamin is an amorphous calcium sodium phosphosilicate that reacts in aqueous environments to release calcium, phosphate and sodium ions over time. This inorganic compound was developed as a bone-regenerative material, but has also shown to be combined with tooth due to its high affinity with collagen, when exposed to saliva emitting phosphate and calcium, through the development of a hydroxyapatite-like mineral layer. Novamin allows phosphate and calcium to be released from the surface. These crystals are known to fill in microscopic surface defects, making teeth stronger, smoother, and less sensitive. This agent also minimizes surface protein precipitation that can limit the ability of chromagens to bind to teeth over time [7].

Roselino et al. [11] reported that dentifrice abrasiveness did not interfere with the ability to remove stains from aged composites. The authors also claimed that staining is material-dependent. Other studies have researched toothbrushing has an effect on optical properties and surface roughness of esthetic dental materials [9, 10]. Toothbrush abrasion however could cause wear of composite resins, most commonly on the buccal surface [14]. As smaller and more homogeneous fillers of a material would reduce the amount of exposed organic matrix, material type, surface-finishing techniques, composite particle size, distribution, and homogeneity might also influence these results, decreasing wear after brushing [23]. The oxidation of amine accelerators in the composite material can cause discoloration, changing hue from a whitish to a yellow appearance. Exogenous sources such as coffee, tea, and nicotine are factors of extrinsic discoloration and these factors may be responsible for visibly detectable or esthetically unacceptable color changes in dental materials or natural tooth [21]. Perceptible color changes were also observed in the present study for some groups after the experimental procedures (Tables 3 and 4).

The extrinsic staining of natural teeth is mainly explained by incorporation of chromogens into the pellicle layer. Other studies have reported that naked enamel will experience little staining, even when exposed continuously to tea solutions for 24 hours or more [21]. Major contributors to staining of dentation are thought to be the beverages, including tea, coffee, and cola. Staining can occur via different mechanisms, for example, discoloration caused by coffee occurs by both the adsorption and absorption of colorants, so the degree of staining varies. Chan et al. [21] investigated the staining potential of beverages on different composite resins and declared that the greatest amount of discoloration occurs during the first week, with results in this time period differing significantly from all succeeding weeks. It is for this reason that, in order to determine the potential for long-term stain retention, an immersion time of one week was chosen in the present study.

In the present study, artificial saliva was used to storing natural teeth and composite specimens before color measurements. The deposition of stains and the subsequent accumulation of artificial saliva act as a matrix, and may result in discoloration [9]. However, the current study only captured the impact of coffee on the salivary pellicle. Other dietary agents that might affect the surfaces of teeth and dental materials and hence staining should also be considered. The action of toothbrushing associated with the use of toothpastes has been shown to be responsible for an increase in surface roughness in composite resins [11]. In the present study, the composites showed perceptible color changes after the staining procedures. This result might be attributable to the simulated brushing, which resulted in varying degrees of roughness. However, using sensitivity-relief toothpastes increased the staining level (except for Group N), which might be due to the possible staining effects of the ingredients in these toothpastes. Since composite specimens resulted in higher ∆E_00_^*∗*^ values than natural teeth, clinicians should be aware of discoloration effect of resin restorative materials, especially when using in the anterior region, where the possible discolorations will be most noticeable.

Dentists often recommend toothbrushes with soft bristles for patients with sensitive teeth. As the present study was designed to test toothpastes for sensitive teeth, the specimens were brushed with soft brushes. There is still controversy in the literature surrounding cleaning and abrasion with hard vs. soft bristles; however, Zimmer et al. [12] demonstrated better cleaning was possible with hard filaments as compared with soft ones. This may have effected the clinically imperceptible discoloration for both natural teeth and composites in the present study, as the soft brushes might not have been able to clean the surfaces sufficiently.

In one study, [10] the roughness and optical stability (color, translucency, and gloss) of microfilled, microhybrid, and nanofilled-composites submitted to simulated toothbrushing over a period of 10 weeks were evaluated. As a result, authors reported that toothbrushing increased the roughness and diminished the gloss of the resins. In the current study microhybrid composite is preferred by authors, because it is the most used resin material type for anterior restorations by the clinicians [34], and it was aimed to evaluate the optical properties of natural anterior human teeth and resin restorative material applicable in the anterior region, where the possible discolorations will be most noticeable.

In Bernardon et al., [18] the effect of desensitizing dentifrices on dentin wear was evaluated and the results showed that all dentifrices cause a progressive increase in surface loss over time. However, no significant differences among dentifrices were observed. Another study demonstrated that, after 10 hours of simulated toothbrushing, ceramic surfaces exhibited no or little damage, while composite materials exhibited surface deterioration [16]. The effect of toothbrushing on the deterioration of resin materials resulted in a rapid increase in the surface roughness, with the extent depending on the material. Results cannot be easily compared, as these studies were conducted on the basis on different design parameters such as loading, number of strokes, toothbrush and toothpaste. However, the results of the present study indicate that using sensitivity-relief toothpastes for up to six months might cause discoloration for both natural teeth and composite materials, and the amount of staining might differ depending on the toothpaste's ingredients.

Limitations of this study include the possible impacts on results of different dietary factors and more frequent oral hygiene procedures. Furthermore, flat-surface composite specimens were used in this study, which may have affected propensity to stain. Color measurements were done using a colorimeter in the present study. However, when measuring the color of translucent dental materials, small aperture colorimeters are prone to the edge-loss effect. Edge-loss effect generally occurs when illumination and color measurement are made through the same window. Because of the translucency of the materials, illuminating light sent from the device can be absorbed, transmitted, scattered, reflected, and displaced in different directions [22]. However, when the specimens were prepared with a greater diameter than the measurement tip diameter, the possible effects of edge-loss can be minimized [31].

As material surface can affect the staining procedure properly, further research is required to evaluate the surface topography of both composites and natural teeth which have been exhibited to brushing with desensitizing toothpastes, using different type of toothbrushes, over a long period.

## 5. Conclusions

Within the limitations of the present study, the following conclusions were drawn.

Desensitizing toothpastes, used in the study for six months, cause color changes below the values of 50:50% AT, before coffee staining.

Usage of desensitizing toothpastes increased the amount of discoloration after storing in coffee for both composites and natural teeth, except for the novamin-containing toothpaste.

For natural teeth and composites, desensitizing toothpastes containing SF, SA, or CPC cause color changes above the values of 50:50% AT, after simulated brushing and coffee staining.

Using novamin-containing toothpastes caused the natural teeth and composites to become brighter and redder, while the other desensitizing toothpastes diminished brightness and increased yellowness.

## Figures and Tables

**Table 1:** Sensitive-relief toothpastes used in the study.

Toothpaste	Manufacturer	Active ingredients
Proexpert Clinic Line Sensitive (SF)	Ipana	Stannous floride (1450 ppm fluoride)
Sensodyne Rapid Relief (SA)	Sensodyne	Strontium acetate (1040 ppm fluoride)
Signal Sensitive Expert (PcH)	Unilever	Potassium citrate, hydroxyapatite (1450 ppm fluoride)
GUM Paroex Sensivital (CPC)	Sunstar GUM	Cetylpyridinium chloride (950 ppm fluoride)
Colgate Sensitive Pro-Relief (A)	Colgate-Palmolive	Arginine (1450 ppm fluoride)
Sensodyne Repair&Protect (N)	Sensodyne	Novamin (450 ppm fluoride)

**Table 2:** The study methodology.

1. Specimen preparation (n=8) (natural tooth/composite)	5. Second color measurements (L_1_, a_1_, b_1_)
↓	↓
2. Storage in artificial saliva (24 hr)	6. Evaluation of ΔE_00_^*∗*^_1_
↓	↓
3. Initial color measurements (L_0_, a_0_, b_0_)	7. Staining with coffee/ brushing each day
↓	↓
4. Simulated brushing	8. Final color measurements (L_2_, a_2_, b_2_)
	↓
	9. Evaluation of ΔE_00_^*∗*^_2_

**Table 3:** Color differences of natural teeth after brushing and staining with coffee.

	Group SF	Group SA	Group PcH	Group CPC	Group A	Group N	Group C
ΔE_00_^*∗*^_1_	1.1 ± 0.3^c^	0.8 ± 0.1^cb^	0.6 ± 0.1^b^	0.8 ± 0.1^cb^	0.8 ± 0.3^cb^	0.6 ± 0.1^b^	0.3 ± 0.2^a^
ΔE_00_^*∗*^_2_	2.6 ± 0.4^ex^	2.1 ± 0.2^fx^	1.7 ± 0.3^dx^	2.5 ± 0.3^ex^	1.6 ± 0.3^dx^	1.0± 0.2^c^	1.1 ± 0.3^cx^

ΔL_1_^*∗*^	1.2 ± 0.3^c^	0.8 ± 0.1^b^	0.7 ± 0.1^b^	0.8 ± 0.3^b^	1.0 ± 0.1^bc^	0.9 ± 0.3^bc^	0.4 ± 0.1^a^
ΔL_2_^*∗*^	2.9 ± 0.3^dx^	2.3 ± 0.1^cx^	1.7 ± 0.3^bx^	2.7 ± 0.2^dx^	2.1 ± 0.1^cx^	1.2 ± 0.2^ax^	1.0 ± 0.1^ax^

Δa_1_^*∗*^	0.2 ± 0.1^a^	0.3 ± 0.2^a^	0.1 ± 0.1^a^	0.2 ± 0.2^a^	0 ± 0.1^a^	0 ± 0.1^a^	0.1 ± 0.2^a^
Δa_2_^*∗*^	0.5 ± 0.3^abx^	0.4 ± 0.1^ax^	0.3 ± 0.1^a^	0.6 ± 0.1^bx^	0.6 ± 0.2^bx^	0.1 ± 0.1^ax^	0.3 ± 0.1^ax^

Δb_1_^*∗*^	1.1 ± 0.4^b^	0.8 ± 0.2^b^	0.5 ± 0.1^a^	1 ± 0.2^b^	0.4 ± 0.2^a^	0.3 ± 0.2^a^	0.3 ± 0.2^a^
Δb_2_^*∗*^	2.6 ± 0.3^cdx^	2.0 ± 0.1^dx^	1.8 ± 0.2^dx^	2.5 ± 0.3^cx^	1.4 ± 0.2^adx^	0.8 ± 0.1^bx^	1.2 ± 0.3^ax^

*Note.* x=significant differences between the ΔE_00_^*∗*^_1_ - ΔE_00_^*∗*^_2_, ΔL_1_^*∗*^ - ΔL_2_^*∗*^, Δa_1_^*∗*^ - Δa_2_^*∗*^, and Δb_1_^*∗*^ - Δb_2_^*∗*^ values (p<0.05); same superscript letters in the same row indicate no significant differences between the groups (p>0.05).

**Table 4:** Color differences of composites after brushing and staining with coffee.

	Group SF	Group SA	Group PcH	Group CPC	Group A	Group N	Group C
ΔE_00_^*∗*^_1_	1.6±0.2^a^	1.2±0.2^b^	1.3±0.1^b^	0.8±0.1^c^	0.7±0.1^c^	0.6±0.1^c^	1.1±0.1^bc^
ΔE_00_^*∗*^_2_	3.1±0.2^ax^	2.7±0.1^acdx^	2.3±0.2^bcdx^	2.8±0.2^acx^	2.6±0.2^acdx^	2.1±0.2^bdx^	2.2±0.2^dx^

ΔL_1_^*∗*^	1.5 ± 0.5^a^	1.3 ± 0.2^a^	1.1 ± 0.4^abc^	0.8 ± 0.1^b^	1.0 ± 0.2^bc^	0.9 ± 0.2^bc^	0.4 ± 0.1^b^
ΔL_2_^*∗*^	3.1 ± 0.4^ax^	2.9 ± 0.3^bx^	1.9 ± 0.3^ax^	3.0 ± 0.2^abx^	2.6 ± 0.4	1.8 ± 0.3^bx^	1.8 ± 0.2^bx^

Δa_1_^*∗*^	0.6 ± 0.2^a^	0.7 ± 0.2^a^	0.3 ± 0.2^b^	0.2 ± 0.1^b^	0 ± 0.2^b^	0 ± 0.3^b^	0.1 ± 0.2^b^
Δa_2_^*∗*^	1.1 ± 0.3^a^	0.4 ± 0.4^bx^	0.6 ± 0.3^bx^	0.8 ± 0.3^abx^	0.8 ± 0.3^abx^	0.4 ± 0.2^bx^	0.4 ± 0.2^bx^

Δb_1_^*∗*^	1.8 ± 0.3^a^	1.1 ± 0.5^bc^	1.5 ± 0.4^ab^	1 ± 0.2^c^	0.4 ± 0.2^d^	0.3 ± 0.2^d^	0.3 ± 0.2^d^
Δb_2_^*∗*^	3.3 ± 0.3^ax^	3.1 ± 0.2^abx^	2.9 ± 0.4^bx^	3.1 ± 0.4^abx^	3.2 ± 0.6^abx^	2.7 ± 0.3^bx^	2.8 ± 0.4^bx^

*Note.* x = significant differences between the ΔE_00_^*∗*^_1_ - ΔE_00_^*∗*^_2_, ΔL_1_^*∗*^ - ΔL_2_^*∗*^, Δa_1_^*∗*^ - Δa_2_^*∗*^, and Δb_1_^*∗*^ - Δb_2_^*∗*^ values (p < 0.05); same superscript letters in the same row indicate no significant differences between the groups (p > 0.05).

## Data Availability

The data used to support the findings of this study are available from the corresponding author upon request.
